# Higher total faecal short-chain fatty acid concentrations correlate with increasing proportions of butyrate and decreasing proportions of branched-chain fatty acids across multiple human studies

**DOI:** 10.1017/gmb.2022.1

**Published:** 2022-03-30

**Authors:** Maria LaBouyer, Grietje Holtrop, Graham Horgan, Silvia W. Gratz, Alvaro Belenguer, Nicola Smith, Alan W. Walker, Sylvia H. Duncan, Alexandra M. Johnstone, Petra Louis, Harry J. Flint, Karen P. Scott

**Affiliations:** 1 Gut Health Group, Rowett Institute, University of Aberdeen, Foresterhill, Aberdeen, Scotland, UK; 2 Biomathematics and Statistics Scotland (BioSS), Aberdeen, Scotland, UK; 3 Nutrigenomics and Fish Growth Endocrinology Group, Institute of Aquaculture of Torre de la Sal, (IATS - CSIC), Castellon, Spain

**Keywords:** Short chain fatty acids, butyrate, branched chain fatty acids, human gut microbiota, faecal pH

## Abstract

Metabolites produced by microbial fermentation in the human intestine, especially short-chain fatty acids (SCFAs), are known to play important roles in colonic and systemic health. Our aim here was to advance our understanding of how and why their concentrations and proportions vary between individuals. We have analysed faecal concentrations of microbial fermentation acids from 10 human volunteer studies, involving 163 subjects, conducted at the Rowett Institute, Aberdeen, UK over a 7-year period. In baseline samples, the % butyrate was significantly higher, whilst % iso-butyrate and % iso-valerate were significantly lower, with increasing total SCFA concentration. The decreasing proportions of iso-butyrate and iso-valerate, derived from amino acid fermentation, suggest that fibre intake was mainly responsible for increased SCFA concentrations. We propose that the increase in % butyrate among faecal SCFA is largely driven by a decrease in colonic pH resulting from higher SCFA concentrations. Consistent with this, both total SCFA and % butyrate increased significantly with decreasing pH across five studies for which faecal pH measurements were available. Colonic pH influences butyrate production through altering the stoichiometry of butyrate formation by butyrate-producing species, resulting in increased acetate uptake and butyrate formation, and facilitating increased relative abundance of butyrate-producing species (notably *Roseburia* and *Eubacterium rectale*).

## Introduction

Short-chain fatty acids (SCFA) are the major products of microbial fermentation of non-digested dietary substrates that reach the human large intestine. They are the main metabolites of microbial fermentation of dietary carbohydrates and fibre, while branched-chain fatty acids (BCFA) are additional products of protein metabolism (Smith and Macfarlane, 1998; Scott et al., 2013; Yao et al., 2016). It has long been recognised that these acids have major impacts on the gut environment, on mucosal absorption and host physiology (Cummings et al., 1978). In addition to supplying energy from dietary fibre via SCFA absorption, these acids have multiple effects on host gene expression and cellular development as inhibitors of histone de-acetylation and through signaling via G-protein coupled receptors (see recent review by Blaak et al., 2020). The effects have been shown to include anti-inflammatory action via the maturation of regulatory T-cells, and the production of hormones that influence satiety (Morrison and Preston, 2016).

The three predominant SCFA products (acetate, propionate and butyrate) differ in their distribution through the host, modes of action and consequences for health (Chambers et al., 2018). Butyrate is the preferred energy source for colonic epithelial cells and has been particularly associated with the maintenance of gut health because of its role in the prevention of colitis and colorectal cancer (Pryde et al., 2002; Hamer et al., 2008; Louis et al., 2014). It is therefore important to determine to what extent, and why, the relative production of these major SCFA in the gut varies within human populations. Since most SCFA produced are rapidly absorbed in the colon, faecal concentrations represent a balance between production and absorption and represent approximately 5–10 percent of the total production (Boets et al., 2017). The higher concentrations in the proximal compared to the distal colon (Cummings et al., 1987) reflect both greater bacterial fermentation (production) at that site and less time for absorption. Transit rate through the colon also affects the amount of absorption and correlates with faecal SCFA concentrations (Lewis and Heaton, 1997; Müller et al., 2020). Nevertheless, many studies have demonstrated diet-associated changes in faecal SCFA concentrations, indicating that they can be a proxy for monitoring overall changes in the balance between production and absorption (Duncan et al., 2007; Boets et al., 2015).

Although some of the earliest literature represents carbohydrate fermentation by anaerobic gut communities as a single balanced equation (Wolin, 1960), it is now clear that fermentation stoichiometry can vary with the species composition of the gut microbiota, with the types of substrate fermented, and with the general gut environment (Flint and Juge, 2015; Louis and Flint, 2017; Reichardt et al., 2018). Thus many factors can potentially influence the relative production rates of acetate, propionate and butyrate *in vivo.* Added to this is the influence of metabolite cross-feeding, as many butyrate-producing bacteria in the colon are also net consumers of acetate (Duncan et al., 2002; Louis and Flint, 2017). A stable isotope study illustrating the systemic availability of colonically administered labelled SCFA showed that 24 per cent of acetate was converted to butyrate (Boets et al., 2017).

We report here an analysis of multiple studies conducted with human volunteers in Aberdeen, UK, in which data are available for SCFA concentrations in human stool samples. This is a valuable dataset with comparable information for a large number of volunteers that can provide significant insights into the function of the human gut microbiota. The results reveal some highly significant relationships, in particular between % butyrate and total SCFA concentrations that build on classic dietary intervention studies involving human volunteers (Cummings et al., 1978; Stephen et al., 1987; Lewis and Heaton, 1997).

## Methods

### Human studies

All the human studies included (Table 1) recruited volunteers locally who did not take antibiotics in the 3 months preceding study participation, nor during the studies, and were conducted according to guidelines laid down in the Declaration of Helsinki, with approval from the appropriate local ethical committee (North of Scotland Research Ethics committee or internal Rowett Institute Human Studies committee). Written consent was obtained from all participants. Samples within studies were collected at the timepoints stated in the respective study protocol. Here, we mainly compare the baseline samples from the volunteers entering the different studies, which represent the free-living population. Any volunteers who participated in more than one study were only considered once by excluding them from datasets in later studies. Details of the dietary regimes followed are detailed in publications relating to individual studies, and are described in the text when appropriate (see Table 1).

**Table 1.** Volunteer studies included in this analysis.

Study ID	*n* ^a^	Females	Males	Fully controlled	Habitual diet data	Bacterial quantification method	pH y/n	SCFA y/n	Energy Intake y/n	BMI range	Reference
778	18	0	18	Yes	No	FISH	No	Yes	Yes	29.6–41.5	Duncan *et al*. (2007)
779	17	0	17	Yes	No	FISH, qPCR	Yes	Yes	Yes	27.9–48.5	Russell *et al*. (2011) and Duncan *et al*. (2008)
780	14	0	14	Yes	No	qPCR	No	Yes	Yes	27.9–51.3	Walker *et al*. (2011), Holtrop *et al*. (2012) and Salonen *et al*. (2014)
782	18	0	18	Yes	No	No	Yes	Yes	Yes	27.8–52.6	Lobley *et al*. (2015) and Gratz *et al*. (2019)
783	20	0	20	Yes	No	qPCR	No	Yes	Yes	26.5–43	Neascu *et al*. (2014)
Plantain	17	12	5	No	Yes	FISH	No	Yes	No	18.3–35.8	Scott *et al*. (this study)
Inulin	12	9	3	No	Yes	qPCR	Yes	Yes	No	19.8–31.5	Fuller *et al*. (2007) and Ramirez-Farias *et al*. (2009)
FruitVeg	38	24	14	No	Yes	qPCR	No	Yes	Yes	17.8–36.6	Duthie *et al*. (2018) and Louis *et al*. (this study)
Oatibix	5	4	1	No	Yes	FISH	Yes	Yes	No	17.6–26.3	Scott *et al*. (this study)
Timebugs	4	2	2	No	Yes	FISH	Yes	Yes	Yes	20.9–26.3	Duncan *et al*. (this study)
Total *n* =	163	51	112								

a *n* = Number of volunteers on each study, followed by number of Females/Males.

Where recorded, energy intake was the average sum of energy (kcal) from carbohydrate, protein, fat and fiber per day based on standardised baseline diets (studies 778 – 783) or by use of a 4-day self-reported food diary (FruitVeg) or weekly averages of daily intake records for Timebugs study.

Abbreviations: BMI, body mass index; FISH, fluorescent in situ hybridisation; qPCR, quantitative polymerase chain reaction.

### Sample processing

Faecal samples were collected and the fresh samples processed within 12 h of collection following standard protocols in place at the Rowett Institute (Duncan et al., 2007; Walker et al., 2011). The number of samples collected varied in the different studies, and the number used in each comparison is indicated at the appropriate point in the results section. Specific methods used for sample processing and analysis are shown in Table S1, illustrating the consistency across all studies.

### SCFA analysis

SCFA analysis was carried out using gas chromatography using derivatised samples as previously described in Richardson et al. (1989). In brief, following derivatisation of the samples using N-tert-butyldimethylsilyl-N-methyltrifluoroacetamide with 2-ethyl butyrate as the internal standard, the samples were analysed using a Hewlett Packard gas chromatograph (GC) fitted with a silica capillary column and using helium as the carrier gas.

### Microbiota analysis

Fluorescent in situ hybridisation (FISH) and quantitative polymerase chain reaction (qPCR) analyses were carried out as detailed in Walker et al. (2005) and Ramirez-Farias et al. (2009), respectively. Any variations on standard protocols are detailed in publications relating to specific individual studies.

### Statistical analysis

Associations between individual SCFA ratios and total SCFA were examined via Pearson’s correlation. All statistical tests were performed using R 3.6.0 (R Foundation for Statistical Computing, Vienna), with significance limits set at *p* < 0.05. Significance of correlations based on multiple observations per volunteer was assessed using a linear mixed model with volunteer as a random effect. Figures were prepared using R version 3.6.3 (R foundation for Statistical Computing, Vienna).

## Results

Data on the concentrations of SCFA in faecal samples were compiled from 10 studies conducted in Aberdeen, UK, between 2006 and 2012. Five of these studies involved obese or overweight male volunteers who were enrolled onto carefully controlled dietary intervention trials looking at the impact of diet upon weight loss. The remaining five studies involved healthy volunteers from the general population who received different supplements to their habitual diets (four studies), while Timebugs was a longitudinal study with no dietary intervention (see Table 1 and previous publications, references provided in Table 1, for details).

**Figure 1:**
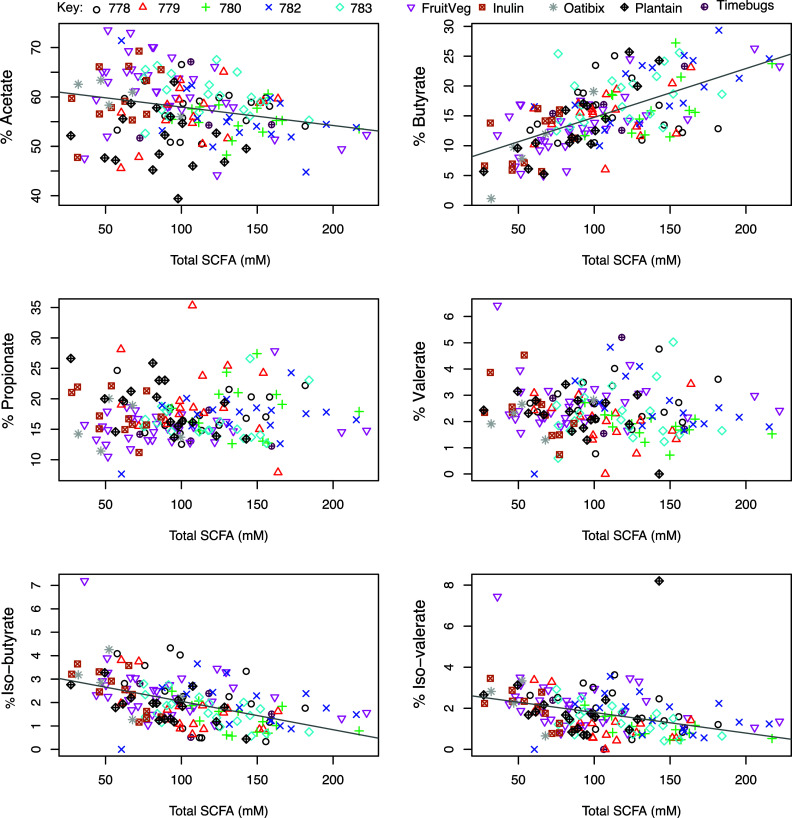
Relationship between total faecal short-chain fatty acid (SCFA) concentrations and proportional abundance (%) of individual fermentation acids for single baseline samples from 158 of the 163 volunteers. Excludes five volunteers with no samples for SCFA analysis. The sum of all SCFA concentrations (Total SCFA, mM) is on the *x*-axis while the percentage of each respective SCFA (individual mM divided by total SCFA concentration in mM) is on the *y*-axis. Pearson’s correlation and linear mixed model analysis gave the following values. Acetate: *r* = −0.247, *p* = 0.002; Propionate: *r* = 0.042, *p* = 0.602; Iso-butyrate: *r* = −0.492, *p* < 0.001; Butyrate: *r* = 0.627, *p* < 0.001; Valerate: *r* = −0.126, *p* = 0.116; Iso-valerate: *r* = −0.373, *p* < 0.001.

We first examined data from baseline samples provided by each volunteer before any major dietary intervention. These corresponded to the subjects’ habitual diets in the five ‘normal weight’ studies, or following a balanced weight maintenance diet for 1–3 days in the case of the five obesity studies. Results from these single samples from 158 different volunteers are shown in Figure 1, in which the percentage of each fermentation acid within the total SCFA pool was plotted against absolute total faecal SCFA concentrations. Three relationships were highly significant (*p* < 0.001). The % butyrate increased with increasing total SCFA concentration (correlation *r* = 0.627, *p* < 0.001) while % iso-butyrate and % iso-valerate decreased with increasing total SCFA concentration (*r* = −0.492 and *r* = −0.373 respectively, *p* < 0.001). There was also a significant negative correlation between the % acetate and total SCFA concentration (*r* = −0.247, *p* = 0.002). The same relationships were also observed when all datapoints (*n* = 502) across the time courses of the various dietary interventions were included (Figure S1), again with the strongest positive correlation between % butyrate and total SCFA (*r* = 0.597, *p* < 0.001). Across the entire dataset, as the total SCFA concentration increased, percentages of acetate, valerate, iso-valerate and iso-butyrate all decreased significantly (*p* < 0.001; Figure S1).

**Figure 2:**
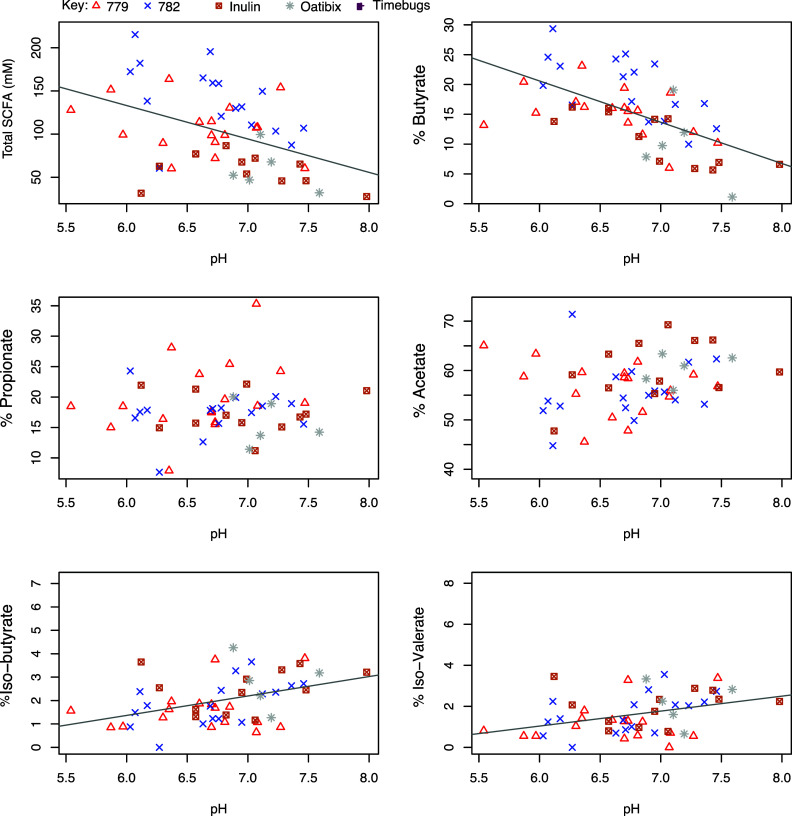
Correlation between faecal pH, short-chain fatty acid (SCFA) and SCFA proportions in baseline samples collected from five volunteer studies (*n* = 51). The pH is on the *x*-axis while total SCFA concentration (mM) or the individual SCFA percentage is on the *y*-axis. Pearson’s correlation and linear mixed model analysis gave the following values. Total SCFA: *r* = −0.425, *p* = 0.002; Propionate: *r* = 0.058, *p* = 0.686; Iso-butyrate: *r* = 0.415, *p* = 0.002; Butyrate: *r* = −0.588, *p* < 0.001; Acetate: *r* = 0.198, *p* = 0.164; Iso-valerate: *r* = 0.377, *p* = 0.006.

Faecal pH measurements were available for samples from five of the studies listed in Table 1. Figure 2 shows the relationship between SCFA concentrations and pH in the baseline samples (*n* = 51). This reveals a significant inverse correlation between total SCFA concentration and faecal pH (*r* = −0.425, *p* =0.002, and specifically between high % butyrate and low faecal pH (*r* = −0.588, *p* < 0.001). In contrast, there was a significant positive correlation between % iso-butyrate (*r* = 0.415, *p* = 0.002) and % iso-valerate (*r* = 0.377, *p* = 0.006) and faecal pH. There was no significant correlation between the % acetate, or % propionate and faecal pH (Figure 2). The strong correlations between decreasing total SCFA concentrations and decreasing % butyrate with increasing pH were also observed when data for all available timepoints (*n* = 198; including dietary intervention periods) were plotted against pH (Figure S2, *r* = −0.503, *p* < 0.001; *r* = −0.602, *p* < 0.001, respectively). Across the full dataset, weak positive correlations were observed between pH and the % propionate (*r* = 0.142, *p* = 0.049) and % acetate (*r* = 0.163, *p* = 0.022), while the strong positive correlation for % iso-butyrate (*r* = 0.245, *p* < 0.001) and pH was still present.

**Figure 3:**
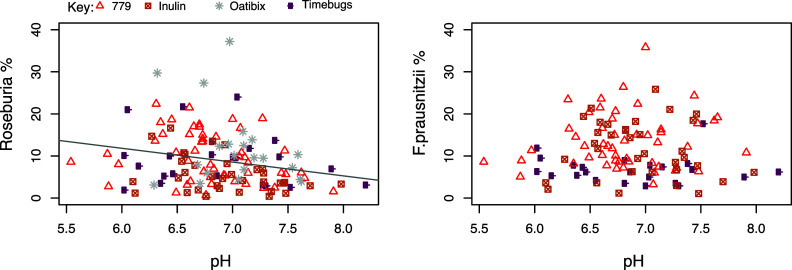
Relationship between faecal pH and proportional abundance (%) of butyrate-producing bacterial groups (*Roseburia*/*E. rectale* and *F. prausnitzii)* within faecal microbiota. Data are based on FISH microscopy counts (Oatibix, Timebugs and 779 study) or quantitative polymerase chain reaction (qPCR) analysis (Inulin study) of all samples from volunteers in four studies (*n* = 130) in the case of *Roseburia*, and three studies (*n* = 109) in the case of *F. prausntizii* (*F. prausnitzii* data were not obtained from the Oatibix study). Pearson’s correlation and linear mixed model analysis gave values of: *Roseburia* % *r* = −0.239, *p* = 0.017; *F. prausnitzii* % *r* = −0.010, *p* = 0.829.

Populations of butyrate-producing bacteria related to *Roseburia* spp. (including *Eubacterium rectale*) were previously shown to decrease in parallel with % butyrate in faecal samples in dietary interventions (Duncan et al., 2007). Here, data from all timepoints for four studies in which pH and microbial composition was measured (*n* = 130; Table 1) showed a significant inverse relationship (*r* = −0.239, *p* = 0.017) between pH and the relative abundance of the *Roseburia* group within the faecal microbiota (Figure 3). No such relationship was found between pH and *Faecalibacterium prausnitzii*, which is another commonly abundant butyrate producing taxon in the human gut, after combining data from three studies (*n* = 109; Figure 3).

**Figure 4:**
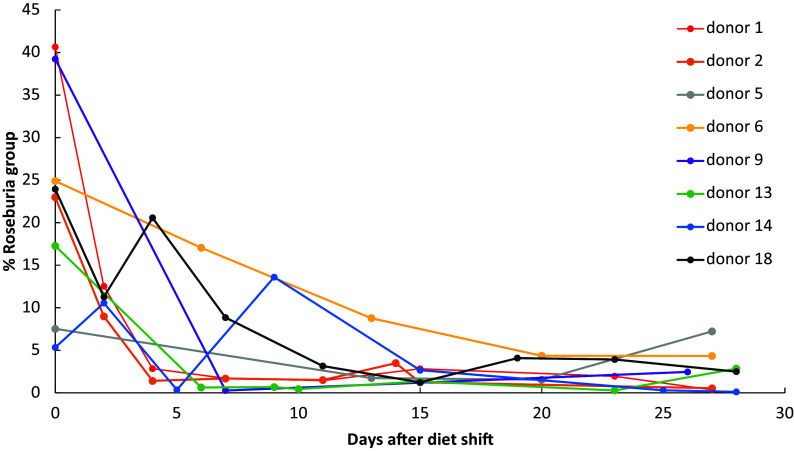
Change in the proportion of *Roseburia/E. rectale* 16S rRNA gene amplicons in faecal samples from overweight volunteers following switch from a maintenance diet to a decreased carbohydrate, weight loss diet. Estimates are from quantitative polymerase chain reaction (qPCR) data from study 779, analysed as described in Hamer et al. (2008) and Ramirez-Farias et al. (2009). The number of days after the diet shift is on the *x*-axis while the % of *Roseburia*/*E. rectale* group 16S rRNA gene calculated as a % of total bacterial 16S rRNA genes is on the *y*-axis. The proportional representation of the *Roseburia* group declined significantly (*p* = 0.008) from five days after the diet shift (non-parametric test of means before and after day 5).

In order to specifically investigate links between dietary carbohydrate consumption and butyrate-producing bacteria, for one of the longitudinal studies (study 779), we followed changes in the population of the *Roseburia* group (as measured by qPCR) over time following the switch from maintenance to a low carbohydrate diet in eight volunteers. These data demonstrate a rapid response to the dietary intervention, with numbers of the *Roseburia* group declining markedly (*p* = 0.008) from 5 days of the shift to the low carbohydrate diet in all volunteers (Figure 4).

**Figure 5:**
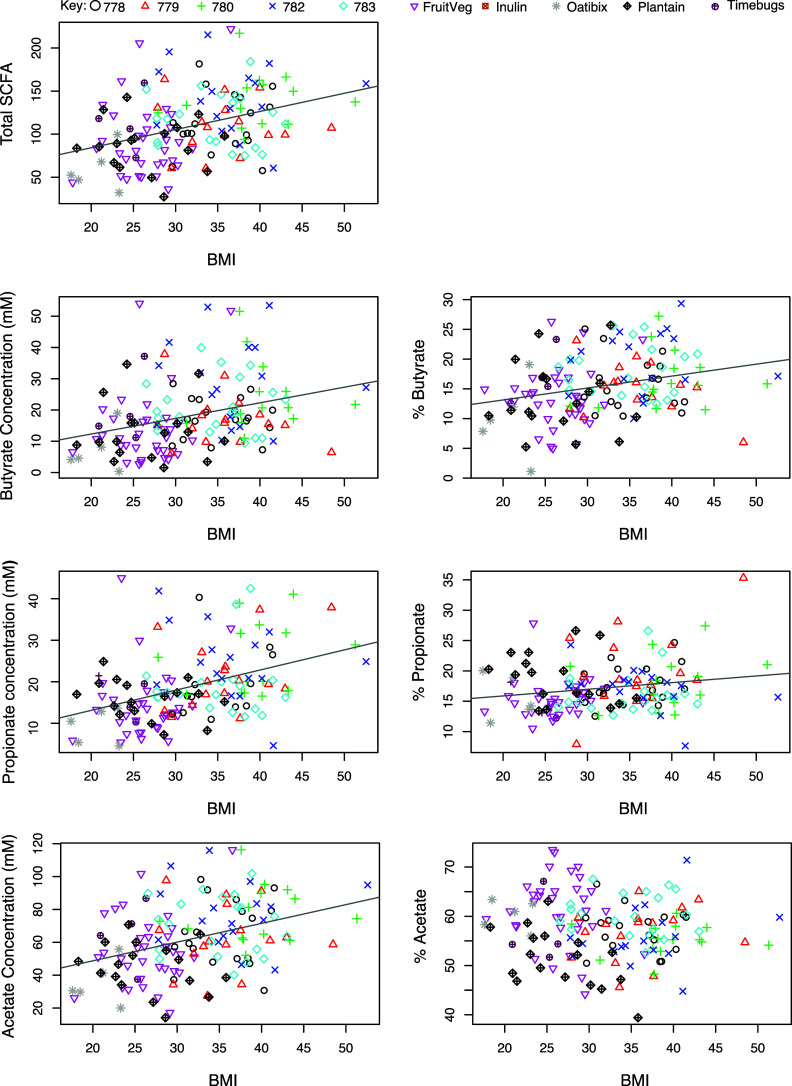
Relationship between total faecal short-chain fatty acid (SCFA) or concentrations of individual SCFA in baseline samples versus body mass index in nine studies where body mass index (BMI) was specifically recorded (*n* = 145). Pearson’s correlation and linear mixed model analysis gave the following values. Total SCFA: *r* = 0.369, *p* < 0.001; Butyrate: *r* = 0.308, *p* < 0.001; % Butyrate: *r* = 0.266, *p* = 0.001; Propionate: *r* = 0.393, *p* < 0.001; % Propionate: *r* = 0.188, *p* = 0.023; Acetate: *r* = 0.369, *p* < 0.001; % Acetate: *r* = −0.112, *p* = 0.179.

Across volunteers from all 10 studies, total faecal SCFA concentrations in baseline samples increased significantly with body mass index (BMI) (*p* < 0.001, Figure 5). We should note that the 5 studies, 778–783, involved only male volunteers who were overweight or obese, while the other studies involved volunteers of both sexes, of mixed BMI (*n* = 145; Table 1). Increased faecal SCFA have been reported previously in obese subjects of both sexes (Schwiertz et al., 2010). Plots of individual SCFAs against BMI showed that concentrations of each of the three major SCFAs (butyrate, acetate and propionate) also all increased significantly with increasing BMI (*p* < 0.001). These significant increases in SCFA concentrations converted into significant positive correlations between increased proportional abundance of butyrate and propionate (*p* = 0.001 and *p* = 0.023 respectively, Figure 5) with increased BMI. Although the mean concentrations of butyrate and total SCFA were significantly lower in females than males (*p* < 0.001; Figure S3A), this appears to reflect the higher mean body mass and energy intakes of male compared to female volunteers involved in these studies (Figure S3B). Total food intake and intake of dietary fibre were highly correlated in these studies (data not shown), making it impossible to distinguish separate relationships between fibre intake and SCFAs.

## Discussion

We reported previously that % butyrate decreased and % iso-valerate and iso-butyrate increased in faecal SCFA in response to reduced carbohydrate intake in small groups of overweight volunteers (Duncan et al., 2007; Russell et al., 2011). The wider analysis presented here of samples donated by 163 individuals from 10 human volunteer studies reveals significant changes in the ratios of different SCFA with increasing total faecal SCFA concentration. Specifically, as the total SCFA concentration increases, the proportion (%) of butyrate increases and % iso-valerate and % iso-butyrate decreases. These relationships were significant both across the baseline samples, and when complete longitudinal data from the full dietary intervention periods were included. The simplest interpretation is that inter-individual variation in the volunteers’ habitual dietary intake results in variation in the delivery of fermentable fibre to the large intestine, which is reflected in the bacterial fermentation products detected in faecal samples.

There are two distinct mechanisms involving colonic pH that could explain the increase in % butyrate with increasing total faecal SCFA concentration. The first is that the stoichiometry of butyrate formation by dominant butyrate-producing bacteria, including *Roseburia* spp. and *Faecalibacterium prausnitzii,* changes with gut luminal pH. These species rely on the butyryl-CoA:acetate CoA-transferase reaction for the final step in butyrate formation that can involve the net uptake of acetate (Duncan et al., 2002). More butyrate is formed, and more acetate taken up, per mol of hexose fermented by pure cultures when the pH is slightly acidic than when it is closer to neutrality (Kettle et al., 2015; Louis and Flint, 2017). This effect appears to account for a 25–50 per cent higher proportion of butyrate among the SCFA products of *in vitro* batch incubations with mixed faecal bacteria observed when the initial pH was 5.5 compared to 6.5 (Reichardt et al., 2018). This effect occurred across a wide range of microbial community composition and was not observed for propionate formation in the same incubations (Reichardt et al., 2018). Since there is evidence that higher SCFA concentrations result in lower pH values in the colon *in vivo* (Bown et al., 1974; Cummings et al., 1978) this stoichiometric shift would increase the % butyrate produced as total SCFA concentrations increase.

pH may also play a crucial role in determining the competition for carbohydrates in the colon. Chemostat studies with human colonic microbiota have shown that major butyrate-producing species compete better for soluble carbohydrates when the pH is slightly acidic than when it is close to neutrality. This is explained by decreased competition between these butyrate-producers and *Bacteroides* species that are more sensitive to acidic pH (Walker et al., 2005; Duncan et al., 2009; Chung et al., 2016). Theoretical modelling was used to demonstrate that the experimental changes in community composition and butyrate formation were consistent and predictable from the behaviour of cultured human colonic bacteria (Duncan et al., 2009; Kettle et al., 2015).

Another possible mechanism is that an increased supply of dietary fibre to the proximal colon promotes the growth and activity of butyrate-producing species, thereby increasing their representation within the microbial community. Evidence for this *in vivo* comes from carefully controlled dietary intervention studies that show a significantly higher proportional representation (and absolute numbers) of butyrate-producing bacteria related to *Roseburia* (including *Eubacterium rectale*) in faecal microbiota from individuals when consuming diets high in fibre compared with diets low in total carbohydrate and fibre (Duncan et al., 2007; Russell et al., 2011). Indeed, a fourfold decrease in the *Roseburia* population was associated with fourfold lower % butyrate (and butyrate concentration) among faecal SCFAs (Duncan et al., 2007). The link between high fibre consumption and higher abundance of the *Roseburia* group has also been reported in other studies (Adamberg et al., 2020), and there is evidence that some of these bacteria specialise in the utilisation of insoluble fibres (Duncan et al., 2016). Fermentable carbohydrates also tend to favour lactate-producing *Bifidobacterium* species. Since several species of colonic bacteria are known to produce butyrate from lactate (Louis and Flint, 2017) this could stimulate butyrate formation indirectly via metabolite cross-feeding.

Iso-butyrate and iso-valerate are products of the fermentation of branched chain amino acids, mainly by *Bacteroides* and *Clostridia* species (Aguirre et al., 2016; Rios-Covian et al., 2020). The % iso-butyrate and iso-valerate among total SCFA is reported to increase with high protein, low carbohydrate diets (Russell et al., 2011; Gratz et al., 2019), while BCFA levels are inversely correlated with consumption of dietary fibre (Rios-Covian et al., 2020). The decrease in % BCFA with increasing total SCFA therefore suggests that high faecal SCFA concentrations are largely attributable to increased carbohydrate fermentation that does not yield BCFA. If we assume that dietary protein is largely digestible (Cummings and Macfarlane, 1997; Van der Wielen et al., 2017), then the delivery of ‘resistant’ dietary protein to the colon is not likely to increase greatly, if at all, on high-fibre diets, while endogenous sources of protein should be largely independent of fibre intake. Therefore, increased fibre intake is expected to lead to fermentation of more non-digestible carbohydrate relative to protein in the large intestine.

Another potential contributor to the observed correlations between higher % butyrate, higher total faecal SCFAs and lower faecal pH is gut transit time. Several studies have shown that gut transit rate can play a major role in determining metabolite concentrations and microbiota profiles. In early studies that used drug or fibre intake to vary gut transit, generally in small groups of volunteers, more rapid whole gut transit was associated with increased faecal SCFA concentrations, increased % butyrate and decreased colonic pH (Cummings et al., 1978; Stephen et al., 1987; El Oufir et al., 1996; Lewis and Heaton, 1997; Abell et al., 2006). More recently, longer gut transit times correlated with higher microbial diversity and species richness (Roager et al., 2016; Vandeputte et al., 2016). Constipated patients across different age-groups appear to have lower total SCFA concentrations, much lower concentrations of butyrate and lower numbers of butyrate-producing bacteria than age-matched healthy controls (Zhuang et al., 2019).

Increased gut transit rate is considered to lead to increased faecal SCFA concentrations for two main reasons (Stephen et al., 1987; Flint, 2011). First, there is evidence that an increase in transit rate through the upper gut may result in less complete digestion of food components, thus increasing the amount of digestive residue arriving in the large intestine and increasing the rate of microbial fermentation (Holgate and Read, 1983; Chapman et al., 1985). Second, more rapid transit is expected to decrease the proportion of SCFA produced by microbial fermentation that is subsequently absorbed across the colonic mucosa. Individuals with slow colonic transit tend to show lower faecal concentrations of SCFA (Müller et al., 2020), and longer average colonic transit times are reported in healthy females than in healthy males (Rao et al., 2009; Wang et al., 2015). The present study was not designed to examine the influence of gender, but we can note that gender-associated differences in the microbiota composition have been linked to BMI (Haro et al., 2016).

In conclusion, inter-individual variation in habitual dietary intakes, in particular fibre intake, are likely to be the main factors accounting for the systematic variation in microbial fermentation and SCFA concentrations observed among human volunteers. The stimulation of butyrate, the main energy source for colonocytes, by decreased colonic pH associated with high microbial SCFA production, noted here, is likely to be a significant factor in the protective effect of fibre consumption against colorectal cancer and certain other bowel diseases.

## Supplementary material

For supplementary material accompanying this paper visit https://doi.org/10.1017/gmb.2022.1. click here to view supplementary material

## Research transparency and reproducibility

The data used to prepare this manuscript were generated from 10 previous studies, many published as primary papers. The combined dataset is available in the Supplementary file entitled ‘faecal SCFA and butyrate study data tables’.
