# Supplementary material for: Higher total faecal short-chain fatty acid concentrations correlate with increasing proportions of butyrate and decreasing proportions of branched-chain fatty acids across multiple human studies
Source: Gut Microbiome (Camb). 2022 Mar 30;3:e2. doi: 10.1017/gmb.2022.1 (PMC11406374; doi:10.1017/gmb.2022.1)
Supplement: Supplementary file 1 [file S2632289722000019sup001.zip › S2632289722000019sup005.docx]

**Supplementary Table 1. Details of methods used for sample collection and analysis relevant to this manuscript for the individual volunteer studies**

| Study ID | n* | Faecal sample  collection | Faecal sample processing method | DNA extraction method |  | Bacterial quantification method^1^ |  | SCFA^2^ |  | Reference |
| --- | --- | --- | --- | --- | --- | --- | --- | --- | --- | --- |
| 778 | 18 | Samples stored 4 °C,  processed same day | Hand homogenisation | n/a |  | **FISH**, 10 probes |  | GC |  | Duncan *et al.,* 2007 |
| 779 | 17 | Samples stored 4 °C,  processed within 5 h | Hand homogenisation | FastDNA Spin  for soil^3^ |  | **FISH**, 10 probes  **qPCR** |  | GC |  | Russell *et al.,* 2011 Duncan *et al.,* 2008 |
| 780 | 14 | Samples stored 4 °C,  processed within 5 h | Hand homogenisation,  Dispomix | FastDNA Spin  for soil^3^ |  | **qPCR**, 16S rRNA  sequencing |  | GC |  | Walker *et al.,* 2011, Holtrop *et al.,* 2012, Salonen *et al.,* 2014 |
| 782 | 18 | Samples stored 4 °C,  processed within 5 h | Homogenisation  (stomacher) | n/a |  | No |  | GC |  | Lobley *et al.,* 2015, Gratz *et al.,* 2019 |
| 783 | 20 | Samples stored 4 °C,  processed within 12 h | Homogenisation,  (stomacher), Dispomix | FastDNA Spin  for soil^3^ |  | **qPCR** |  | GC |  | Neacsu *et al.,* 2014, Louis *et al.,* (this study) |
| Plantain | 17 | Samples stored 4 °C,  processed within 12 h | Hand homogenisation,  Dispomix | n/a |  | **FISH**, 10 probes |  | GC |  | Scott *et al.,* (this study) |
| Inulin | 12 | Samples stored 4 °C,  aliquoted within 12 h | Aliquots stored at -20°C | FastDNA Spin  for soil^3^ |  | **qPCR** |  | GC |  | Fuller *et al.,* 2007, Ramirez-Farias *et al.,* 2009 |
| FruitVeg | 38 | Samples stored 4 °C,  processed within 12 h | Homogenisation,  (stomacher), Dispomix | FastDNA Spin  for soil^3^ |  | **qPCR** |  | GC |  | Duthie *et al.,* 2018, Louis *et al.,* (this study) |
| Oatibix | 5 | Samples stored 4 °C,  processed within 12 h | Hand homogenisation,  Dispomix | n/a |  | **FISH**, 10 probes |  | GC |  | Scott *et al.,* (this study) |
| Timebugs | 4 | Samples stored 4 °C,  processed within 5 h | Hand homogenisation,  Dispomix | n/a |  | **FISH**, 10 probes |  | GC |  | Duncan *et al.,* (this study) |

*n = Number of volunteers recruited on each study. Total n = 163. n/a – not applicable, no DNA extraction for these studies.

1 Bacterial quantification methods used for comparisons in this paper are shown in bold. The FISH method is described in Walker *et al.,* 2005, and the qPCR method in Ramirez-Farias *et al.,* 2009.

2 SCFA and BCFA quantification was done using Gas Chromatography (GC) following the method described by Richardson *et al.,* (1989).

3 FastDNA^TM^ Spin kit for Soil DNA extraction from MP Biomedicals. DNA extracted from fresh samples (779, 780), or from Dispomixed samples stored frozen at -70 °C in PBS/30% glycerol (1:2 wt/vol ratio; 783, FruitVeg). For the inulin study, DNA was extracted from faecal aliquots stored frozen at -20 °C.
