# Supplementary figures and images for: Higher total faecal short-chain fatty acid concentrations correlate with increasing proportions of butyrate and decreasing proportions of branched-chain fatty acids across multiple human studies
Source: Gut Microbiome (Camb). 2022 Mar 30;3:e2. doi: 10.1017/gmb.2022.1 (PMC11406374; doi:10.1017/gmb.2022.1)

Fig S1 Key: ○ 778 △ 779 + 780 × 782 ◇ 783

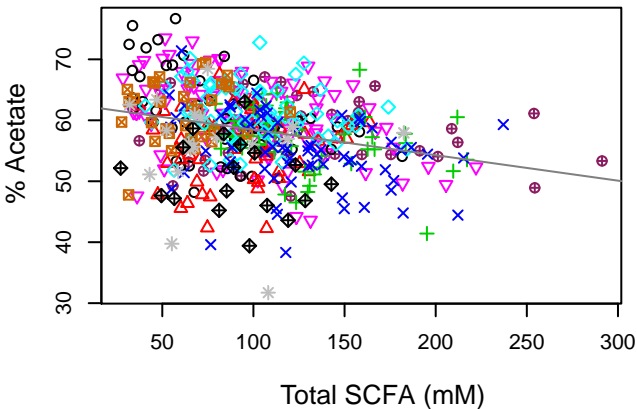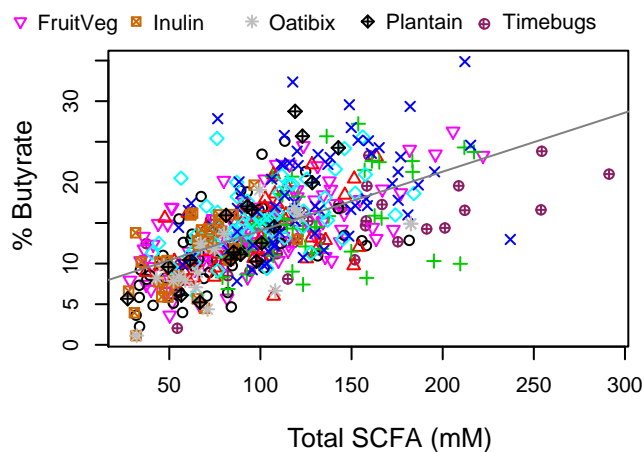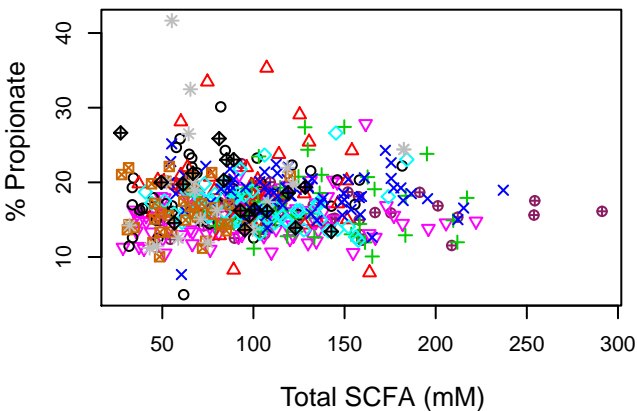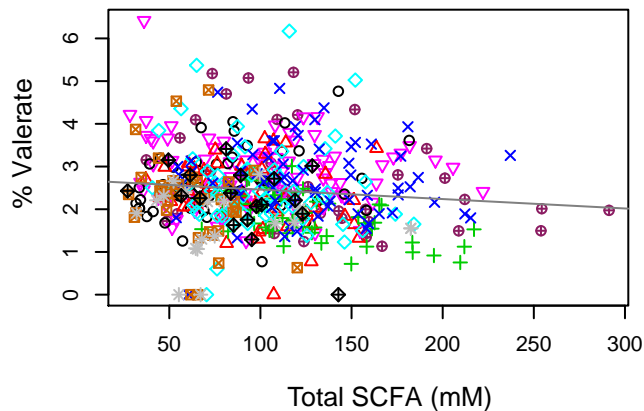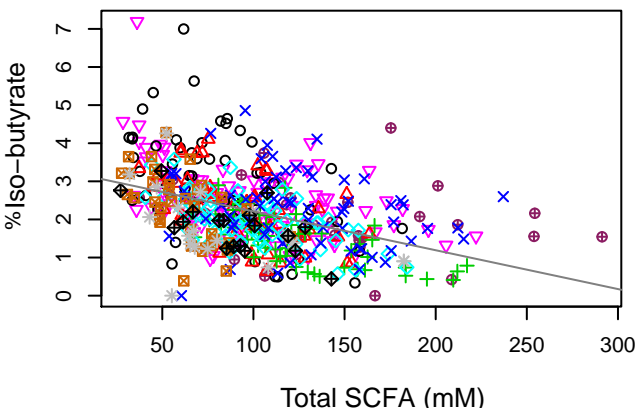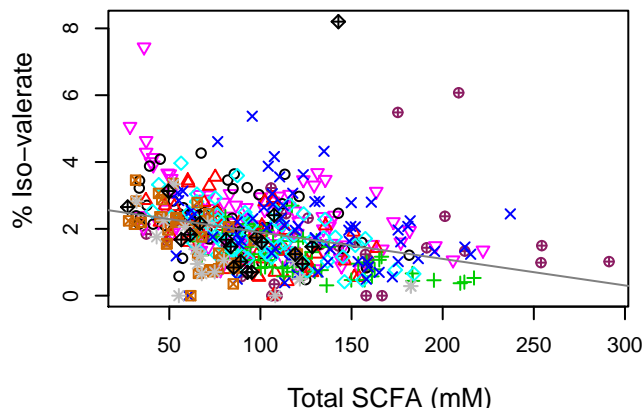

Supplement: Supplementary file 1 [file S2632289722000019sup001.zip › S2632289722000019sup001.pdf]

Fig S3B

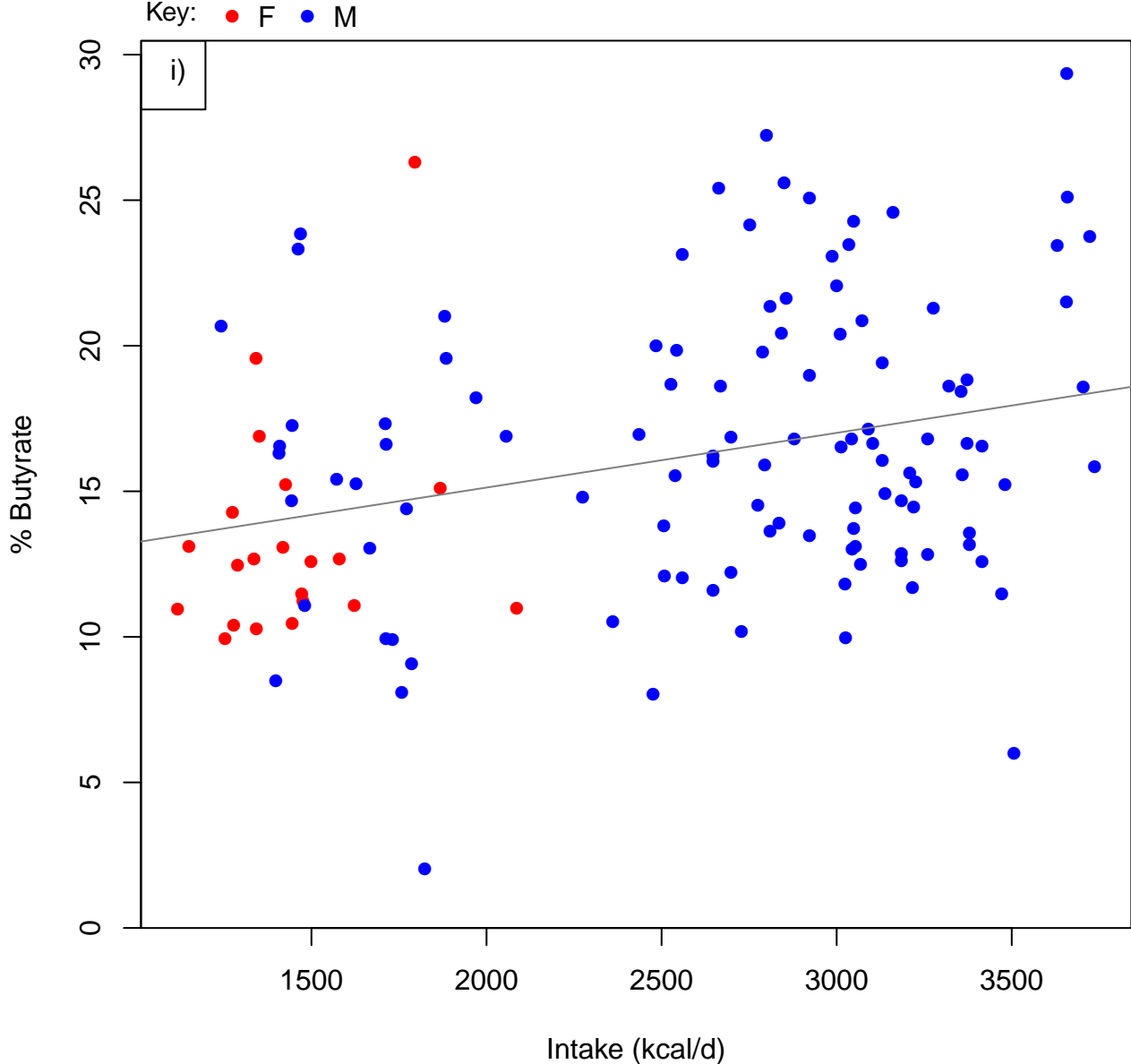

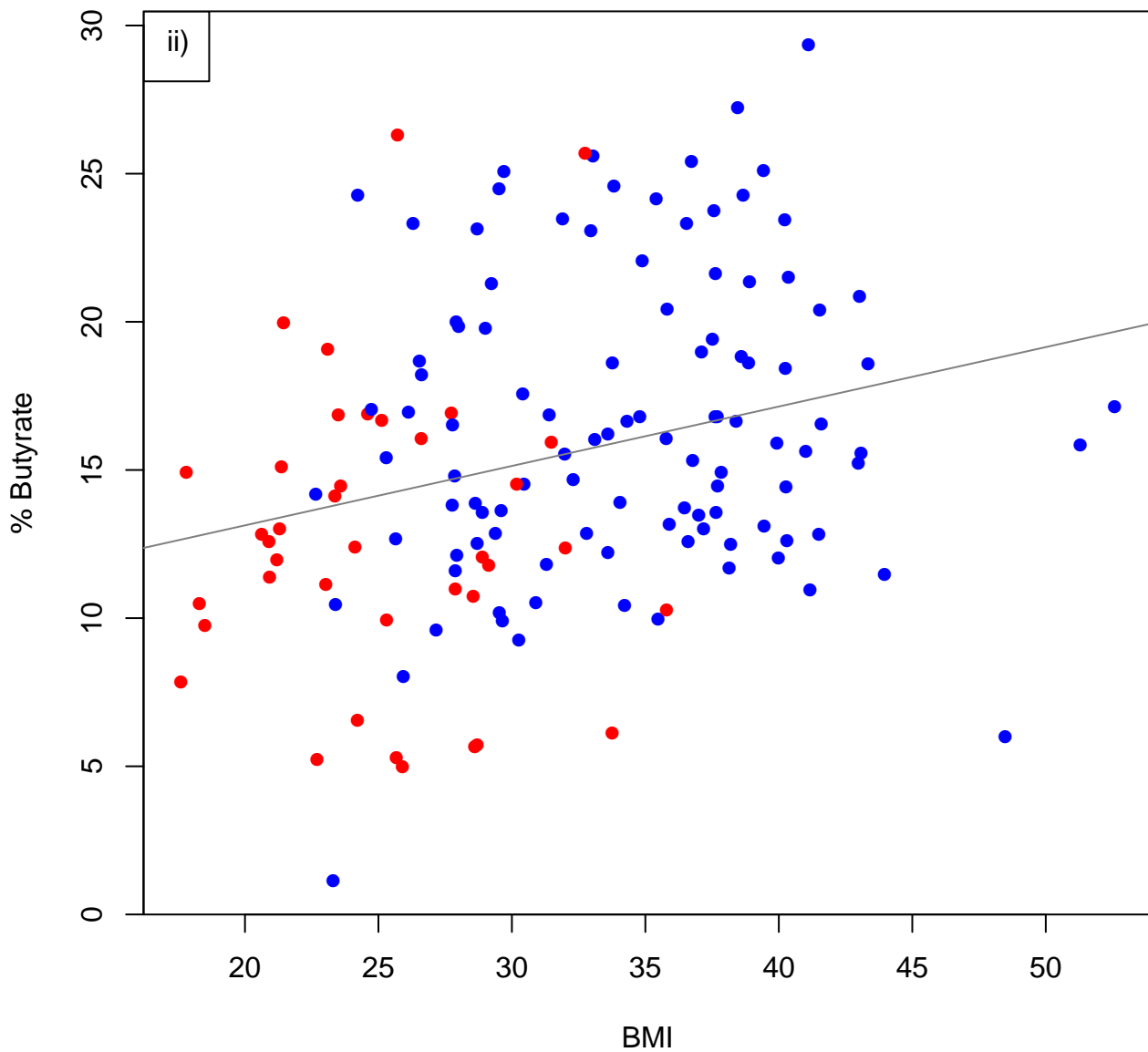

Supplement: Supplementary file 1 [file S2632289722000019sup001.zip › S2632289722000019sup002.pdf]

## Slide 1
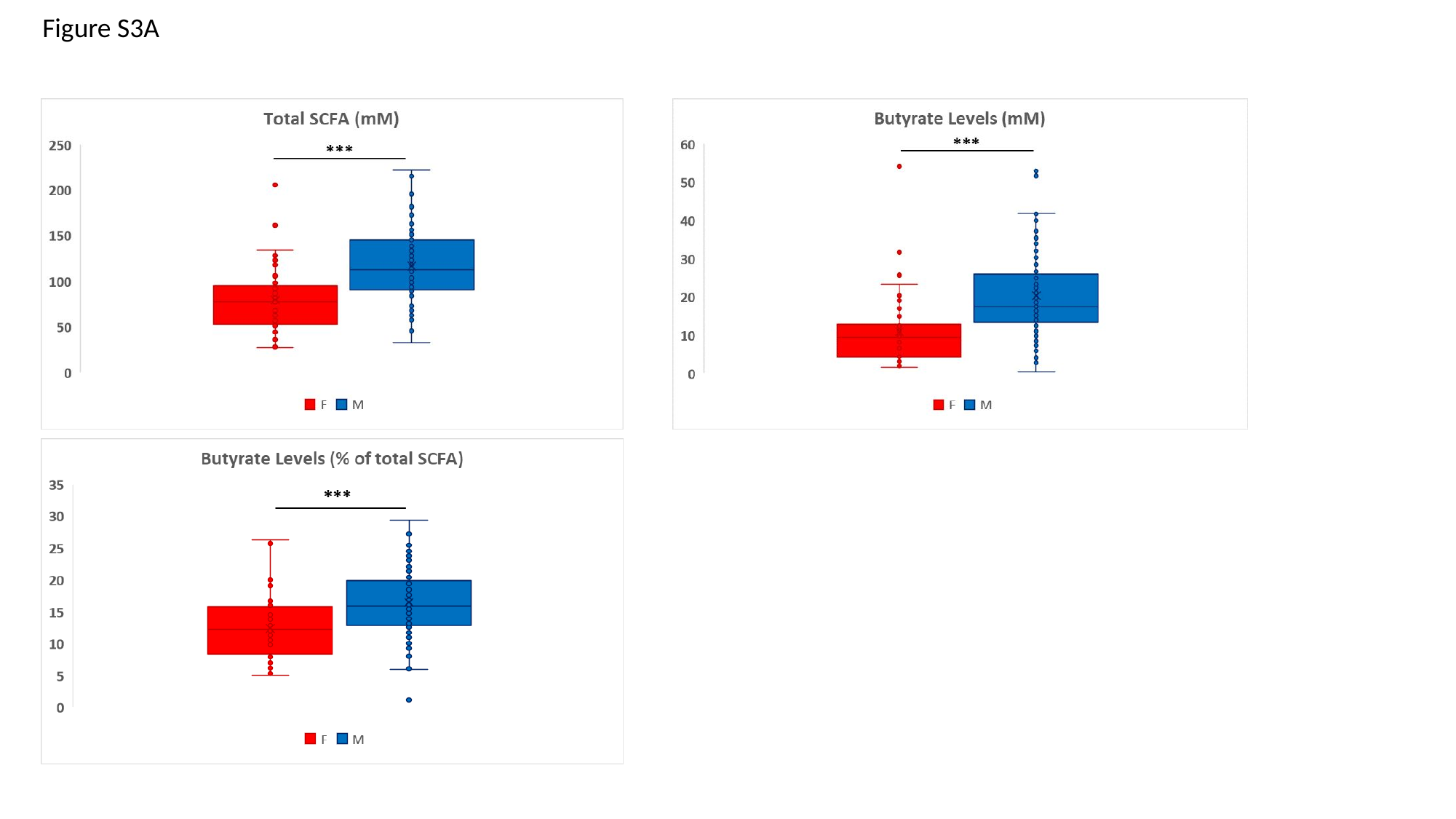

Figure S3A

Supplement: Supplementary file 1 [file S2632289722000019sup001.zip › S2632289722000019sup003.pptx]

**Fig S2** Key:  $\triangle$  779  $\times$  782  $\square$  Inulin  $\ast$  Oatibix  $\oplus$  Timebugs

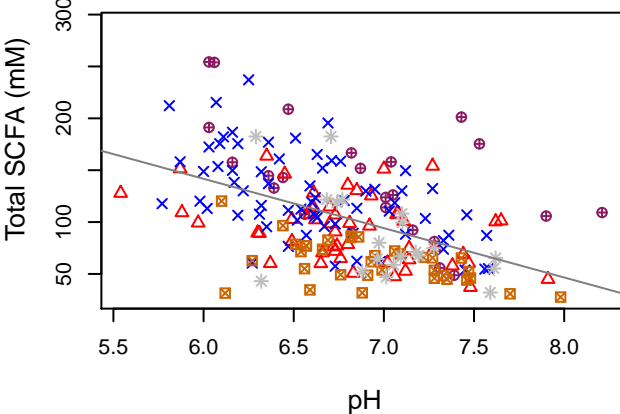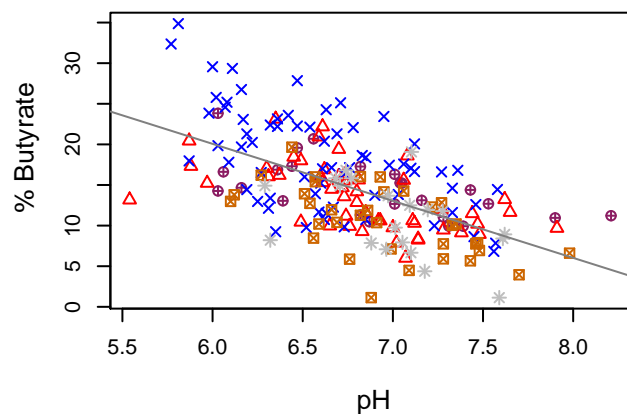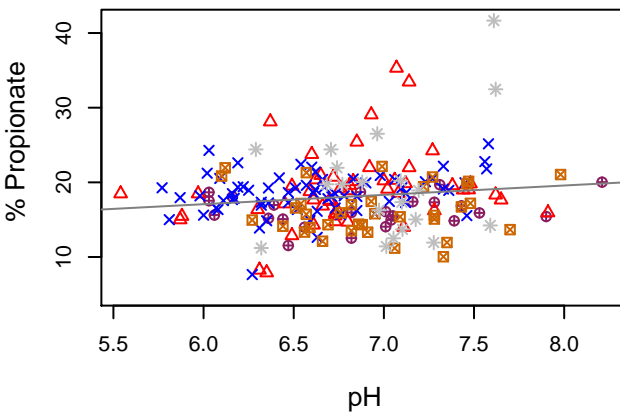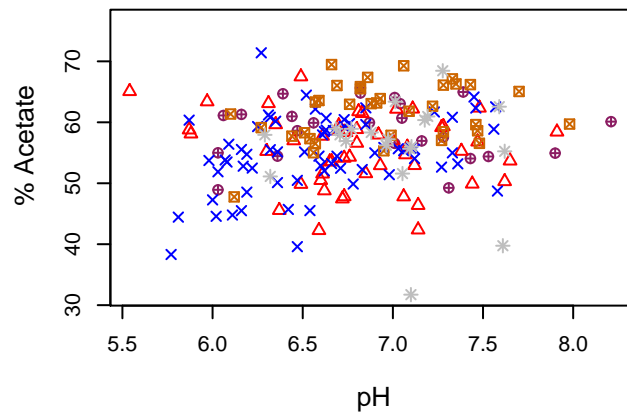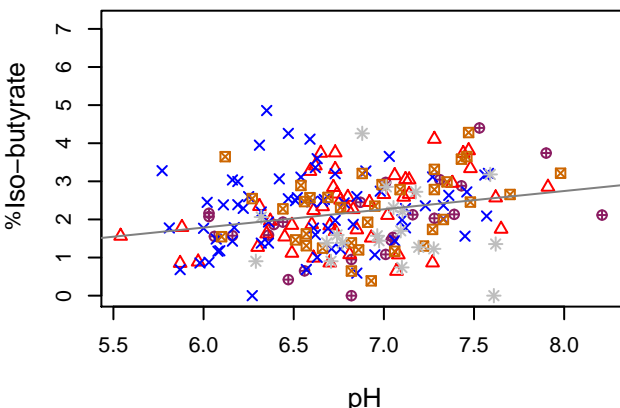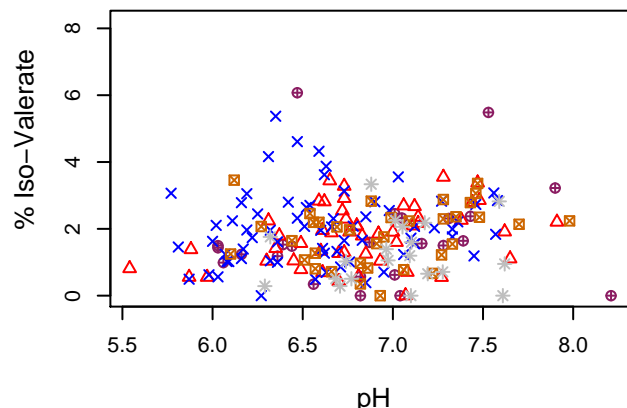

Supplement: Supplementary file 1 [file S2632289722000019sup001.zip › S2632289722000019sup006.pdf]
